# Antihyperglycemic treatment in patients with type 2 diabetes in Italy: the impact of age and kidney function

**DOI:** 10.18632/oncotarget.18816

**Published:** 2017-06-28

**Authors:** Sandro Gentile, Pamela Piscitelli, Francesca Viazzi, Giuseppina Russo, Antonio Ceriello, Carlo Giorda, Piero Guida, Paola Fioretto, Roberto Pontremoli, Felice Strollo, Salvatore De Cosmo, The AMD-Annals Study Group

**Affiliations:** ^1^ Department of Clinical and Experimental Medicine, University of Campania, Naples, Italy; ^2^ IRCSS Casa Sollievo della Sofferenza – Unit of Internal Medicine, Scientific Institute, San Giovanni Rotondo, Italy; ^3^ Università degli Studi, IRCCS Azienda Ospedaliera Universitaria San Martino-IST, Istituto Nazionale per la Ricerca sul Cancro, Genova, Italy; ^4^ Department of Clinical and Experimental Medicine, University of Messina, Messina, Italy; ^5^ Insititut d’Investigacions Biomèdiques August Pi i Sunyer (IDIBAPS) and Centro de Investigación Biomédica en Red de Diabetes y Enfermedades Metabólicas Asociadas (CIBERDEM), Barcelona, Spain; ^6^ U.O. Diabetologia e Malattie Metaboliche, Multimedica IRCCS, Milano, Italy; ^7^ Diabetes and Metabolism Unit, ASL Turin 5, Turin, Italy; ^8^ Statistical Consultant for Associazione Medici Diabetologi (AMD), Rome, Italy; ^9^ Department of Medicine, University of Padova, Padova, Italy; ^10^ Endocrinology and Metabolism, Elle-di, Rome, Italy; ^11^ Associazione Medici Diabetologi (AMD), Rome, Italy

**Keywords:** type 2 diabetes mellitus, nephropathy, age, antihyperglycemic treatment, eGFR

## Abstract

We describe AHA utilization pattern according to age and renal function in type 2 diabetes mellitus (T2DM), in real-life conditions.

The analysis was performed using the data set of electronic medical records collected between 1 January and 31 December, 2011 in 207 Italian diabetes centers. The study population consisted of 157,595 individuals with T2DM. The AHA treatment regimens was evaluated. Kidney function was assessed by eGFR, estimated using the CKD-EPI formula. Other determinations: HbA1c, blood pressure (BP), low- density lipoprotein (LDL-c), total and high density lipoprotein cholesterol (TC and HDL-c), triglycerides (TG) and serum uric acid (SUA). Quality of care was assessed through Q score.

The proportion of subjects taking metformin declined progressively across age quartiles along with eGFR values, but remained high in oldest subjects (i.e. 54.5 %). On the other hand, the proportion of patients on secretagogues or insulin increased with aging (i.e. 54.7% and 37% in the fourth age quartile, respectively). The percentage of patients with low eGFR (i.e. <30 ml/min/1.73m2) taking either metformin or sulphonilureas/repaglinide was particularly high (i.e. 15.3% and 34.3% respectively).

In a large real-life cohort of T2DM, metformin or sulphonylureas/repaglinide, although not recommended, are frequently prescribed to elderly subjects with severe kidney disease.

## INTRODUCTION

Diabetes has been estimated to account for approximately 1.5 million deaths in 2012, with more than 80% of diabetes-related deaths in low- and middle-income countries [1]. Lifestyle modification and glucose-lowering drug treatment are the mainstay of therapy to prevent and delay diabetes-related complications [2–3].

Even though a large number of antihyperglycemic agents are approved for type 2 diabetes (T2DM), major T2DM treatment guidelines suggest metformin as first-line drug treatment, and, if glycemic control is not achieved, the addition of a second drug is recommended [2–3].

Accordingly, in real-life conditions the majority of people with T2DM take metformin alone or in combination, although prescription is not always appropriate with regards to age and renal function limitations [4–6]. In fact, while the cardiovascular benefits associated to the use of metformin have been described, a careful assessment of kidney function is necessary prior to prescribe this drug as it is primarily eliminated via the kidney [7]. More recently, limitations on the use of this drug in individuals with mild or moderate impairment of renal function have been disputed [8–10], because of poor evidence on a specific safety threshold and the lack of clear-cut evidence supporting increased risk of complications (especially lactic acidosis) in the presence of mild or moderate renal impairment. Nonetheless, it is agreed upon that this drug should not be used in the presence of severe kidney dysfunction, i.e. GFR below 30 ml/min/1.73m^2^ [2–3].

Age also needs to be taken into account when choosing antihyperglycemic agents in the clinical setting. This is mainly due to a well known reduction in GFR with aging which may foster the risk of severe hypoglycemic events [11]. To date, only few large studies have investigated the impact of age and impaired kidney function on the use of antihyperglycemic drugs, mainly metformin, in real-life clinical conditions [10, 12–13].

In this context, the large database of the AMD Annals initiative [14] provides a unique opportunity to analyse prescription patterns in Italy and correlate them with the quality of care, assessed through a validated score (Q score).

Therefore, aim of this report was to assess antihyperglycemic treatment, mainly focused on metformin use, in a large sample of patients with T2DM, according to age and kidney function in real-life conditions.

## RESULTS

Clinical features of the whole study sample are reported in Table 1. Overall, the mean age of the participating patients was 68±11 years, 56.7% patients were males and the mean duration of diabetes was 11±9 years. Mean BMI was 30±5 Kg/m^2^. Glycemic control, as well as, lipid parameters and BP levels were fairly good, being mean HbA1c, LDL-c and BP values 7.2± 1.3%, 101±33 mg/dL and 137±18/7±89 mmHg, respectively. Mean eGFR was 76±21 mL/min/1.73m^2^. In Table 1 we describe also participants’ clinical characteristics according to age quartiles. Older participants had longer duration of diabetes; lower BMI, waist circumference and triglyceride levels, as well as, higher HDL-c. Systolic BP and antihypertensive treatment rate increased with age, while the percentage of current smokers decreased. It is worth noting that mean HbA1c was 7.1% in patients in the fourth quartile (mean age 81 years), thus indicating that almost half of patients within this class had HbA1c values below 7.0% and therefore were, very likely, overtreated.

**Table 1:** Clinical characteristics of the whole sample and divided according to age quartiles

	All	<62 years	62-69 years	70-75 years	>75 years	p
	n=157595	n=39407	n=39394	n=39404	n=39390	
Male sex (n)	89290 (56.7%)	24674 (62.6%)	23345 (59.3%)	22140 (56.2%)	19131 (48.6%)	<0.001
Age (years)	68±11	53±7	65±2	72±2	81±4	-
Former smokers (n)	25875 (29.3%)	6207 (25.6%)	7355 (32.3%)	6741 (31.4%)	5572 (28.0%)	<0.001
Current smokers (n)	14793 (16.7%)	6605 (27.2%)	4238 (18.6%)	2617 (12.2%)	1333 (6.7%)	<0.001
Age at DM diagnosis (years)	56±12	45±9	54±8	59±9	65±11	<0.001
Known duration of diabetes (years)	11±9	8±7	10±8	13±9	15±11	<0.001
HbA1c (%)	7.2±1.3	7.3±1.5	7.2±1.3	7.2±1.2	7.2±1.2	<0.001
BMI (Kg/m^2^)	30±5	30±6	30±5	29±5	28±5	<0.001
BMI Men (Kg/m^2^)	29±5	30±5	30±5	29±4	28±4	<0.001
BMI Women (Kg/m^2^)	30±6	31±7	31±6	30±6	29±5	<0.001
Waist circumference (cm)	104±13	104±14	104±13	103±12	102±12	<0.001
Waist circumference Men (cm)	104±12	105±13	105±12	104±12	104±11	<0.001
Waist circumference Women (cm)	102±13	103±15	103±13	102±13	101±12	<0.001
Triglycerides (mg/dL)	137±90	151±118	137±82	132±84	128±69	<0.001
HDL (mg/dL)	50±14	48±14	50±14	51±14	52±15	<0.001
HDL Men (mg/dL)	47±13	45±13	47±13	48±13	48±14	<0.001
HDL Women (mg/dL)	54±15	53±15	54±14	54±15	55±15	<0.001
LDL (mg/dL)	101±33	106±34	100±33	99±32	100±33	<0.001
Non-HDL (mg/dL)	128±37	135±40	127±37	124±36	125±36	<0.001
Systolic BP (mmHg)	137±18	132±17	137±18	139±19	140±19	<0.001
Diastolic BP (mmHg)	78±9	80±10	78±9	77±9	76±9	<0.001
Pulse pressure (mmHg)	59±16	53±14	59±15	62±16	64±17	<0.001
Albuminuria (n)	45387 (28.8%)	9843 (25.0%)	10645 (27.0%)	11535 (29.3%)	13364 (33.9%)	<0.001
Microalbuminuria (n)	35801 (22.7%)	7989 (20.3%)	8392 (21.3%)	8995 (22.8%)	10425 (26.5%)	<0.001
Macroalbuminuria (n)	9586 (6.1%)	1854 (4.7%)	2253 (5.7%)	2540 (6.4%)	2939 (7.5%)	<0.001
Serum creatinine (mg/dL)	0.98±0.54	0.86±0.41	0.93±0.50	1.01±0.55	1.10±0.63	<0.001
eGFR (mL/min/1.73 m^2^)	76±21	93±17	80±17	72±18	61±19	<0.001
Retinopathy (n)	22250 (14.1%)	4314 (10.9%)	5729 (14.5%)	6259 (15.9%)	5948 (15.1%)	<0.001
Antihyperglycemic treatments - Lifestyle (n)	8229 (5.2%)	2114 (5.4%)	2145 (5.4%)	2150 (5.5%)	1820 (4.6%)	<0.001
Antihypertensive treatment (n)	112424 (71.3%)	21788 (55.3%)	28342 (71.9%)	30543 (77.5%)	31751 (80.6%)	<0.001
Treatment with ACE-Is/ARBs (n)	95821 (60.8%)	18940 (48.1%)	24518 (62.2%)	26103 (66.2%)	26260 (66.7%)	<0.001
Lipid-lowering treatment (n)	90690 (57.5%)	19866 (50.4%)	24362 (61.8%)	24674 (62.6%)	21788 (55.3%)	<0.001
Treatment with statins (n)	83342 (52.9%)	17464 (44.3%)	22392 (56.8%)	22947 (58.2%)	20539 (52.1%)	<0.001
Aspirin (n)	35284 (22.4%)	5514 (14.0%)	8872 (22.5%)	10253 (26.0%)	10645 (27.0%)	<0.001
eGFR<60 mL/min/1.73 m^2^ (n)	35166 (22.3%)	1879 (4.8%)	5335 (13.5%)	10113 (25.7%)	17839 (45.3%)	<0.001
HbA1c ≥7% (n)	79889 (51.4%)	20147 (52.0%)	19292 (49.7%)	19675 (50.6%)	20775 (53.5%)	<0.001
Total cholesterol (mg/dL)	177±39	182±40	176±38	175±37	176±38	<0.001
Triglycerides ≥150 mg/dl	45467 (30.9%)	13530 (36.3%)	11713 (31.5%)	10610 (28.8%)	9614 (26.7%)	<0.001
HDL <40M <50F mg/dL (n)	52108 (36.0%)	14797 (40.3%)	13057 (35.7%)	12076 (33.4%)	12178 (34.4%)	<0.001
LDL ≥100 mg/dL (n)	69295 (48.1%)	19715 (54.4%)	16990 (46.7%)	16209 (44.8%)	16381 (46.4%)	<0.001
Blood Pressure ≥140/85 mmHg (n)	71462 (53.5%)	15552 (45.8%)	18069 (53.5%)	19204 (57.1%)	18637 (58.0%)	<0.001
Treatment with fibrates (n)	4588 (2.9%)	1652 (4.2%)	1210 (3.1%)	1011 (2.6%)	715 (1.8%)	<0.001
Metformin (n)	108234 (68.7%)	30389 (77.1%)	29574 (75.1%)	26798 (68.0%)	21473 (54.5%)	<0.001
Sulphonylureas/Repaglinide (n)	76869 (48.8%)	16450 (41.7%)	18871 (47.9%)	19996 (50.7%)	21552 (54.7%)	<0.001
Acarbose (n)	4613 (2.9%)	937 (2.4%)	1099 (2.8%)	1233 (3.1%)	1344 (3.4%)	<0.001
Glitazones (n)	4280 (2.7%)	1250 (3.2%)	1263 (3.2%)	1090 (2.8%)	677 (1.7%)	<0.001
Insulin (n)	48831 (31.0%)	10754 (27.3%)	11017 (28.0%)	12489 (31.7%)	14571 (37.0%)	<0.001

Mean±SD or absolute frequency (percentage). ACE-Is=angiotensin converting enzyme-inhibitors, ARBs=angiotensin II receptor antagonists, BMI=body mass index, BP=blood pressure, eGFR=estimated glomerular filtration rate, HbA1c=glycated hemoglobin, HDL =high-density lipoprotein cholesterol, LDL =low-density lipoprotein cholesterol. Patients’ missing data: age at diagnosis and duration of diabetes in 8435 (5.4%), BMI in 14918 (9.5%), Waist circumference in 111567 (70.8%), HbA1c in 2291 (1.5%), total cholesterol in 8127 (5.2%), triglycerides in 10293 (6.5%), HDL-c in 12812 (8.1%), LDL-c in 13495 (8.6%), Non-HDL in 13799 (8.8%), serum uric acid in 81772 (51.9%), GGT in 75760 (48.1%), AST/GOT in 47264 (30%), ALT/GPT in 42967 (27.3%), blood pressure in 24106 (15.3%), and smoking status in 69213 (43.9%). The p values refer to significance of mixed regression models (linear for continuous and logistic for categorical variables) with age quartiles as dependent variables.

As expected, serum creatinine increased and eGFR declined progressively as a function of age. Prevalence of patients with eGFR <60 ml/min/1.73m2 increased across age quartiles form 4.8% in the first to 45.3% in the forth quartile. The proportion of both micro- and macroalbuminuria also significantly increased with age (Table 1).

Antihyperglycemic drugs utilization rate in the whole population and across the age quartiles is also reported in Table 1. Patients taking metformin decreased with age, being 77.1% in the lowest quartile and 54.5% in the highest quartile. Use of pioglitazone (the only thiazolidinedione available in Italy) also decreased across age quartiles and eGFR classes, while treatment with sulphonilureas/repaglinide or insulin increased from the lowest to the highest quartile.

The quality of care, as indicated by Q score, was similar across age quartiles (Supplementary Figure 1).

In Table 2 the clinical features of our population are described as stratified according to eGFR classes. Age, duration of diabetes, triglyceride levels, presence of retinopathy, prevalence rates of micro- and macroalbuminuria, as well as, of antihypertensive, lipid-lowering and antiplatelet treatments increased along with eGFR decrease. However, although metformin utilization decreased in parallel with eGFR values, it is worth to underline that 617 (15.3%) patients with eGFR below 30 ml/min/1.73m2 were kept on metformin (alone or in combination with other oral hypoglycaemic agents or insulin). Similarly sulphonilureas/repaglinide utilization also decreased in patents with low eGFR but a large percentage of patients with eGFR <30 ml/min/1.73m^2^ was still taking sulphonilureas/repaglinide. Finally, insulin utilization rate increased with decreased eGFR as expected, and in fact 68.4% of patients with eGFR <30 ml/min/1.73m2 was on insulin treatment.

**Table 2:** Clinical characteristics of the whole sample and divided according to classes of estimated Glomerular Filtration Rate

	GFR>90	GFR 60-90	GFR 30-60	GFR<30	Overall
	n=47254	n=75175	n=31137	n=4029	p
Male sex (n)	28595 (60.5%)	43080 (57.3%)	15712 (50.5%)	1903 (47.2%)	<0.001
Age (years)	59±10	70±9	75±8	76±9	<0.001
Former smokers (n)	7612 (26.9%)	12657 (30.5%)	4975 (30.3%)	631 (29.9%)	<0.001
Current smokers (n)	7083 (25.0%)	5858 (14.1%)	1658 (10.1%)	194 (9.2%)	<0.001
Age at DM diagnosis (years)	50±11	58±11	60±12	59±13	<0.001
Known duration of diabetes (years)	9±8	12±9	14±10	17±11	<0.001
HbA1c (%)	7.3±1.4	7.2±1.3	7.3±1.3	7.3±1.3	<0.001
BMI (Kg/m^2^)	30±6	29±5	30±5	30±5	<0.001
BMI Men (Kg/m^2^)	29±5	29±5	29±5	29±5	<0.001
BMI Women (Kg/m^2^)	31±6	30±6	30±6	31±6	<0.001
Waist circumference (cm)	103±13	103±12	105±12	107±13	<0.001
Waist circumference Men (cm)	104±13	104±12	106±12	108±13	<0.001
Waist circumference Women (cm)	102±14	102±13	104±13	105±13	<0.001
Triglycerides (mg/dL)	135±97	132±84	149±94	163±96	<0.001
HDL (mg/dL)	50±14	51±14	49±14	46±15	<0.001
HDL Men (mg/dL)	47±13	48±13	45±13	42±13	<0.001
HDL Women (mg/dL)	54±15	55±15	52±15	49±16	<0.001
LDL (mg/dL)	103±33	101±33	99±34	98±34	<0.001
Non-HDL (mg/dL)	129±38	126±37	128±38	130±41	<0.001
Systolic BP (mmHg)	134±18	138±18	139±19	139±20	<0.001
Diastolic BP (mmHg)	79±9	78±9	77±10	75±10	<0.001
Pulse pressure (mmHg)	56±15	60±16	62±17	63±18	<0.001
Albuminuria (n)	11246 (23.8%)	19681 (26.2%)	12026 (38.6%)	2434 (60.4%)	<0.001
Microalbuminuria (n)	9493 (20.1%)	16038 (21.3%)	8877 (28.5%)	1393 (34.6%)	<0.001
Macroalbuminuria (n)	1753 (3.7%)	3643 (4.8%)	3149 (10.1%)	1041 (25.8%)	<0.001
Serum creatinine (mg/dL)	0.72±0.13	0.91±0.15	1.30±0.25	2.93±2.17	-
eGFR (mL/min/1.73 m^2^)	99±8	77±9	48±8	22±7	-
Retinopathy (n)	5281 (11.2%)	10168 (13.5%)	5759 (18.5%)	1042 (25.9%)	<0.001
Antihyperglycemic treatments - Lifestyle (n)	2424 (5.1%)	4414 (5.9%)	1280 (4.1%)	111 (2.8%)	<0.001
Antihypertensive treatment (n)	27506 (58.2%)	54777 (72.9%)	26553 (85.3%)	3588 (89.1%)	<0.001
Treatment with ACE-Is/ARBs (n)	23520 (49.8%)	46837 (62.3%)	22714 (72.9%)	2750 (68.3%)	<0.001
Lipid-lowering treatment (n)	24760 (52.4%)	44003 (58.5%)	19287 (61.9%)	2640 (65.5%)	<0.001
Treatment with statins (n)	22797 (48.2%)	40737 (54.2%)	17447 (56.0%)	2361 (58.6%)	<0.001
Aspirin (n)	7679 (16.3%)	17615 (23.4%)	8788 (28.2%)	1202 (29.8%)	<0.001
eGFR<60 mL/min/1.73 m^2^	0 (0.0%)	0 (0.0%)	31137 (100.0%)	4029 (100.0%)	-
HbA1c ≥7%	23573 (50.6%)	37212 (50.2%)	16963 (55.3%)	2141 (54.1%)	<0.001
Total cholesterol (mg/dL)	179±39	177±38	176±39	176±43	<0.001
Triglycerides ≥150 mg/dl (n)	12929 (29.0%)	20049 (28.4%)	10869 (38.0%)	1620 (45.2%)	<0.001
HDL <40M <50F mg/dL (n)	15516 (35.2%)	22818 (33.0%)	11907 (42.5%)	1867 (53.5%)	<0.001
LDL ≥100 mg/dL (n)	22213 (51.0%)	32993 (47.7%)	12568 (45.1%)	1521 (43.8%)	<0.001
Blood Pressure ≥140/85 mmHg (n)	20000 (48.7%)	35448 (55.5%)	14283 (56.3%)	1731 (54.8%)	<0.001
Treatment with fibrates (n)	1170 (2.5%)	2049 (2.7%)	1218 (3.9%)	151 (3.7%)	<0.001
Metformin (n)	37429 (79.2%)	54434 (72.4%)	15754 (50.6%)	617 (15.3%)	<0.001
Sulphonylureas/Repaglinide (n)	21684 (45.9%)	38060 (50.6%)	15742 (50.6%)	1383 (34.3%)	<0.001
Acarbose (n)	1267 (2.7%)	2096 (2.8%)	1120 (3.6%)	130 (3.2%)	<0.001
Glitazones (n)	1437 (3.0%)	2056 (2.7%)	725 (2.3%)	62 (1.5%)	<0.001
Insulin (n)	12007 (25.4%)	20693 (27.5%)	13375 (43.0%)	2756 (68.4%)	<0.001

Mean±SD or absolute frequency (percentage). Overall p value refers to the significance of model with eGFR in the four categories as explanatory variable. Adjusted p value refers to significance of regression coefficient of each variable in a mixed linear model with continuous eGFR as dependent variable. Legend as in Table 1.

The quality of care, as indicated by Q score, was similar across eGFR classes (Supplementary Figure 1).

When grouping the whole sample according to antihyperglycemic treatment (Supplementary Table 1), patients on insulin (alone or in combination with metformin) turned out to be older and had a longer duration of disease, a lower eGFR and a higher prevalence of micro-macroalbuminuria as compared to those taking either metformin alone or other antihyperglycemic agents.

**Figure 1:**
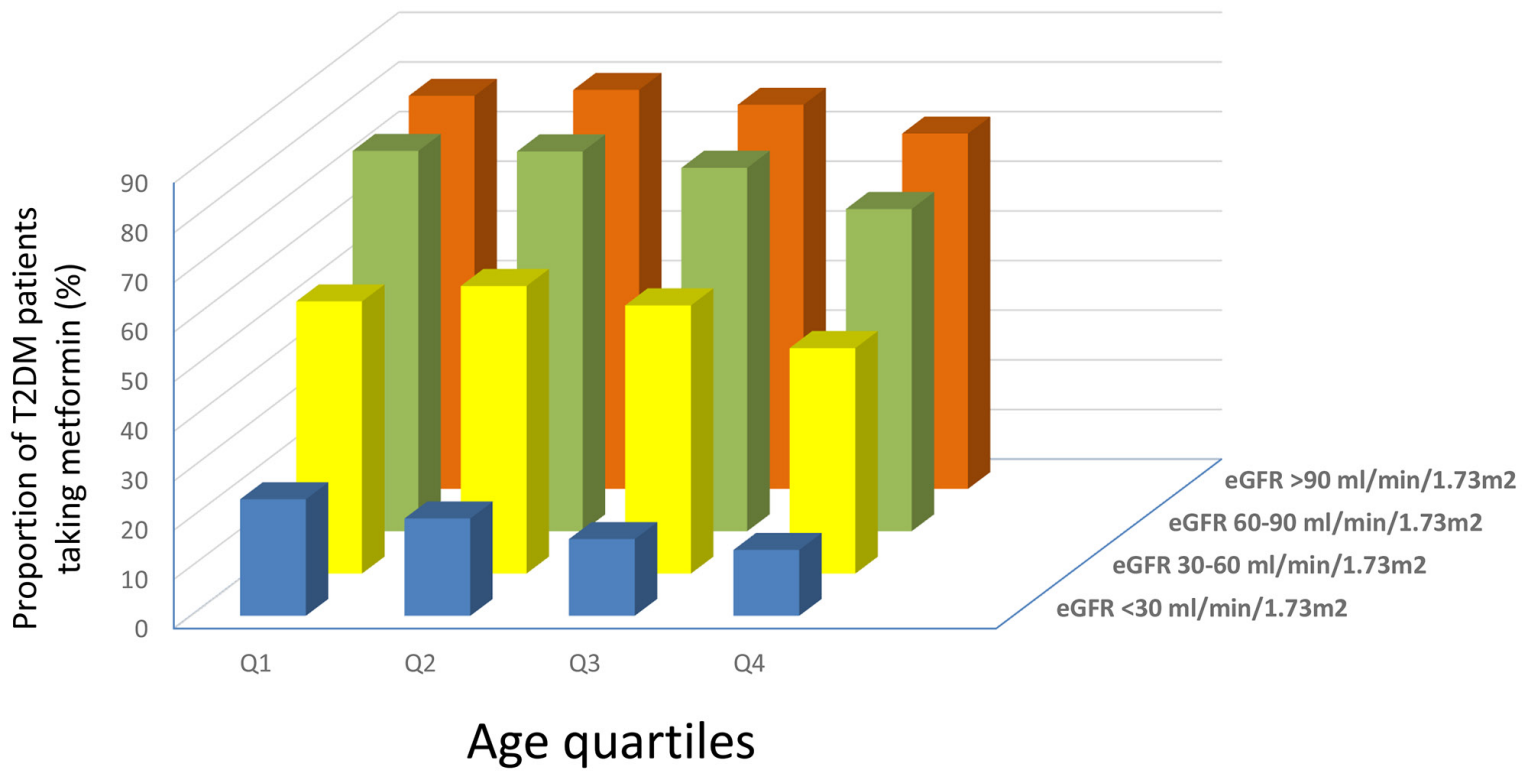
Proportion of T2DM patients taking metformin according to age quartiles and eGFR classes

Then we analysed the distribution of antihyperglycemic agents according to both age and eGFR categories it appeared evident, although unexpected, that metformin and sulphonilureas/repaglinide were largely used in elderly patients in spite of very low eGFR (i.e. eGFR <30 ml/min/173m2) (Supplementary Table 2 and Figure 1).

## DISCUSSION

In this report, we assessed prescription patterns of AHA, mainly focused on metformin, according to age and kidney function in a large sample of patients with T2DM attending diabetes centers in Italy, in real-life conditions.

T2DM is a major health problem for the aging population and, therefore, older people (i.e. > 65 years) are highly prevalent among those attending diabetes centers, being in our series more than 50%. Age should be taken into account when choosing the most appropriate AHA, by keeping an eye not only on target HbA1c [2–3, 15], which often requires to be slightly higher than usual, but also, on progressive GFR decline occurring in people aged 65+.

It’s worth emphasizing that about half of patients in the highest age quartile (i.e. median age: 80 years) had HbA1c levels below 7.0%. Thus, although a less stringent glycemic control is usually suggested in these patients [15], our findings could suggest the presence of a possibly overtreatment in real-life conditions.

A little proportion of our patients was on pioglitazone treatment. Given the well know side effects (i.e. fluid retention, heart failure, fractures), although not contraindicated, its use decreased in elderly and among patients with low or very low eGFR.

As expected, elderly persons had a lower eGFR. In this subgroup of patients, a large proportion (i.e. more than 50%) was treated with sulphonilureas/repaglinide and thus exposed to an increased risk of hypoglycemic events. In fact, a recent survey, investigating in a real-life condition the risk of severe hypoglycemia in 29,485 sulphonilurea treated diabetic patients shown as it was related to older age and decreased eGFR [16]. Older age and diabetic complication, together with diminished food intake, alcohol abuse, use of other medication and concomitant infection, were also the main causes of hospital admission-required hypoglycemia in an observational study including 16,865 T2DM patients from Capital Region of Denmark taking sulphonilureas [17].

Mainly based on data from the UKPDS sub-study related to overweight patients [18], nowadays international treatment guidelines recommend metformin as first-line AHA in patients with T2DM [2–3]. Despite no firm consensus on that, several observational studies support the concept that metformin is effective, as well as, devoid of any increased risk of acidosis, also in patients with significant degree of renal impairment (i.e. eGFR between 60 and 30 ml/min/1.73m2) [19–22]. In fact, an observational study on 51,675 Swedish people with T2DM found no increased risk of acidosis in metformin users with eGFR of 30-45 ml/min/1.73m2 compared with non-users [10]. More recently, a systematic review by Crowley et al. [9] further confirmed metformin to be associated with improvements in clinical outcomes in patients with moderate CKD, thus supporting the recent changes in metformin labeling.

Consequently, to prevent patients from missing beneficial effects of metformin, some health agencies, including the National Institute for Health and Clinical Excellence [2–3], suggest initiation of that drug also in individuals with GFR 46 to <60 ml/min/1.73m^2^ and continuation with additional caution and dose reduction whenever GFR declines to 30-45 ml/min/1.73m^2^.

This position was also confirmed by Inzucchi et al. in a recent systematic review of 65 studies investigating upon the risk of lactic acidosis associated to metformin use. They concluded for a less strict approach to metformin treatment might be chosen, although with caution, in patients with T2DM and mild or moderate chronic kidney disease entangling lower doses and careful follow-up of kidney function [8]. These Authors also strongly suggested to refrain from using metformin in patients with GFR below 30 ml/min to avoid the risk of both lactic acidosis and of increased mortality previously shown to be associated with metformin use in patients having serum creatinine concentrations greater than 530 μmol/L [12].

Although the use of metformin in our sample declined with age, the frequent inappropriate use of this drug in the elderly clearly stands out from our data. In fact, 13.4% (n=309) of the oldest patients with eGFR below 30 ml/min/1.73m^2^ were on metformin (alone or in combination with insulin), and thus at high risk for major complications.

Epidemiological data show suboptimal adherence to different guidelines, which have recommended various kidney function thresholds for metformin restriction in CKD. Our results are in keeping with data from Huang et al. who retrospectively reviewed metformin-treated patients with T2DM admitted to major teaching Hospitals in Australia. They reported that about 31% of these patients received the drug inappropriately, given the presence of contraindications. Kidney failure (i.e. GFR below 30 ml/min/1.73m^2^) was one of the most frequent contraindication [21]. In a study of 83,850 US veterans ≥ 65 years of age with creatinine clearance ranging 15–49 ml/min, metformin was among the 3 medications altogether accounting for 76% of renally misprescribed medications among patients with 30–49 ml/min creatinine clearance rate [22] and, according to the previously quoted systematic review [8], among patients with kidney-related contraindication, as many as one-third were still prescribed metformin.

The awareness of risks associated with metformin misuse is increasing. In fact, a recent survey by Koro C et al. analyzing oral antidiabetic drug utilization rates by 1,462 patients with T2DM and chronic kidney disease from the US NANHES IV database, found that 43.4% was taking OADs (24.0% were on sulphonilureas) but no patient with stage 4 and 5 CKD was taking metformin [13].

The reason why the inappropriate use of metformin is quite diffuse is beyond the aim of this survey. However, the evidence that not all physicians follow clinical practice guidelines [23] and that therapeutic inertia is still an issue [24] could account, at least in part, for metformin misuse. Furthermore, the barriers to start insulin therapy especially in elderly may have also contributed [25]. In addition, the fact that quality of care delivered to the patients did not differ according to age or eGFR classes allows us to rule out any clinical approach inequalities.

Our study has some limitations as well as several strengths. Among the former, we need to say that the data were collected in the 2011, when the use of new innovative antihyperglycemic agents such as DPP-4 inhibitors, GLP-1 agonist or Glifozin was still trivial. In this regard. it is anyway worth to underline that these new classes of drugs are still underused in Italy [26]. Second, we have no information on metformin dosage and duration of treatment. On the other hand, we should mention the large size of the study cohort and the homogeneous geographical distribution of the recruiting centers, which certainly contribute to make the study population a good representation of real-life clinical practice. Furthermore, as some drugs sharing similar pharmacologic mechanisms (i.e. Sulphonylureas and Repaglinide) were pooled together in our database, we were unable to carry out separate analyses for each individual drug class.

In conclusion, although recent guidelines have taken a less stringent stance about contraindications to metformin treatment, clinical risk associated to the use of this drug remains high and should be avoided in the presence of severe CKD. Herewith reported and discussed data indicating the persistence of a significant degree of inappropriateness in the prescription of this drug, call to action for implementing more suitable use of antihyperglycemic drugs, especially in older patients with kidney dysfunction.

## METHODS

### Study setting, study patients and data sources

In the present report we analyzed a large cohort of patients with T2DM followed-up at 207 diabetes centers in Italy among those affiliated to the Italian Association of Clinical Diabetologists initiative aiming to investigate the use of antihyperglycemic treatment, according to age and kidney function. The centers participated in the study are homogeneously distributed throughout the country. The analysis was performed using the data set of electronic medical records collected between 1 January and 31 December 2011. For the purpose of the analysis, we considered only patients who were ≥18 years old and with data about estimated GFR (eGFR) and albuminuria. The study population consisted of 157,595 individuals with T2DM.

### Data collection

Data from all participating centers were collected and centrally analyzed anonymously. The results were internally approved by the AMD Annals scientific committee. The diagnosis of T2DM was made at participating Diabetes Centers according to the American Diabetes Association 2003 criteria. This initiative includes measuring and monitoring HbA1c, blood pressure, low- density lipoprotein, total and high density lipoprotein cholesterol, triglycerides and serum uric acid by high standard auto-analyzers in public laboratories successfully participating in nationwide quality control programs. The use of specific classes of drugs (metformin, other AHA, statins and anti-hypertensive agents) was also evaluated. Kidney function was assessed by serum creatinine and urinary albumin excretion measurements. GFR was estimated for each patient using a standardized serum creatinine assay and the CKD-EPI formula [27]. To be included in the study, the patients had to have at least one measurement of serum creatinine, with concordant eGFR values, in the 3 months prior to study entry. Increased urinary albumin excretion was diagnosed as: i) microalbuminuria if urinary albumin concentration was >30 and ≤300 mg/l, or if UAE rate was >20 and ≤200 μg/min, or if urinary albumin-to-creatinine ratio (ACR) was >2.5 mg/mmol in men and >3.5 mg/mmol in women and ≤30 mg/mmol in both genders; ii) macroalbuminuria if urinary albumin concentration was >300 mg/l, or if UAE rate was >200 μg/min, or if ACR was >30 mg/mmol in both genders. Albuminuria indicated patients with either micro- or macroalbuminuria. At each participating center, all patients underwent physical examination and BP measurements according to a standardized protocol. Information on the presence of diabetic retinopathy was also available.

Quality of care was assessed through the Q score, which was developed as part of the study QuED and subsequently validated in the study QUASAR [28].

### Statistical analysis

Data are given as mean values ± standard deviation (SD); categorical variables are described as frequencies and percentages. Mixed regression models, with diabetes clinics fitted as random effect to consider possible differences in data across centres, were used to compare patients’ characteristics by groups. Continuous and categorical variables were analyzed, respectively, by linear and logistic mixed regression models. P values of <0.05 were considered statistically significant. The analyses were made using STATA software, Version 14 (StataCorp, College Station, Texas).

## SUPPLEMENTARY MATERIALS FIGURE AND TABLES




