# Supplementary material for: Antihyperglycemic treatment in patients with type 2 diabetes in Italy: the impact of age and kidney function
Source: Oncotarget. 2017 Jun 28;8(37):62039–48. doi: 10.18632/oncotarget.18816 (PMC5617484; doi:10.18632/oncotarget.18816)
Supplement: Supplementary file 1 [file oncotarget-08-62039-s001.pdf]

Antihyperglycemic treatment in patients with type 2 diabetes in Italy: the impact of age and kidney function

SUPPLEMENTARY MATERIALS

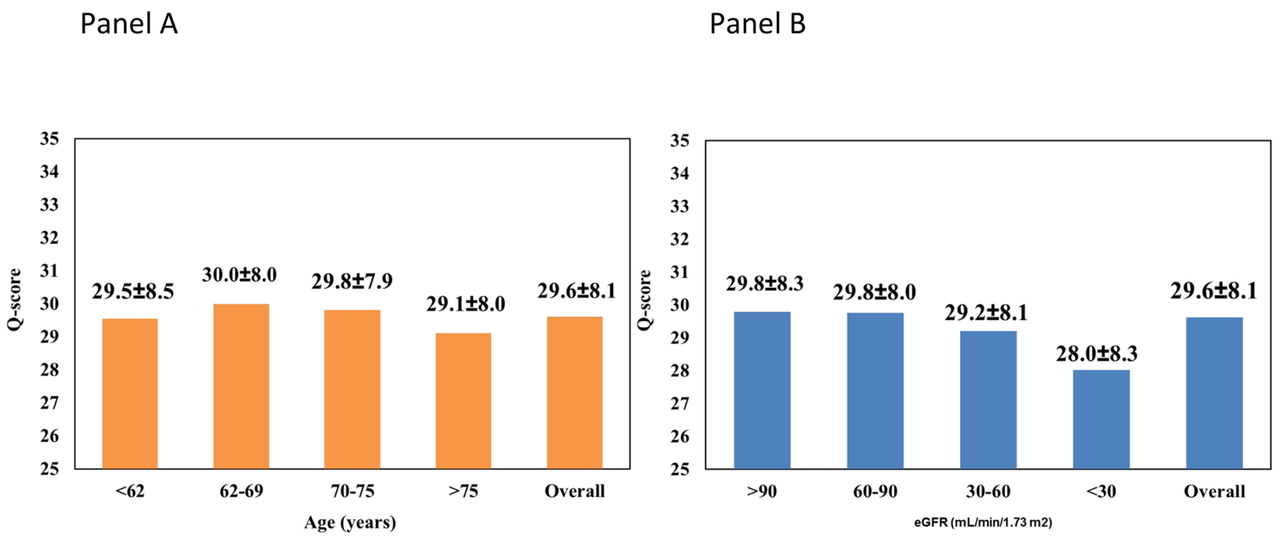

Supplementary Figure 1: Q score values according to age quartiles and eGFR classes.

Supplementary Table 1: Antihyperglycemic treatment regimens in the whole sample and divided according to age quartile and estimated Glomerular Filtration Rate (eGFR) classes

Antihyperglycemic treatments	Age quartiles					p
	All	<62 years	62-69 years	70-75 years	>75 years	
	n=157595	n=39407	n=39394	n=39404	n=39390	
Lifestyle	8229 (5.2%)	2114 (5.4%)	2145 (5.4%)	2150 (5.5%)	1820 (4.6%)	<0.001
Lifestyle eGFR Category 1	2424 (5.1%)	1263 (5.0%)	810 (5.4%)	304 (5.5%)	47 (4.3%)	0.125
Lifestyle eGFR Category 2	4414 (5.9%)	782 (6.5%)	1137 (6.0%)	1410 (5.9%)	1085 (5.3%)	<0.001
Lifestyle eGFR Category 3	1280 (4.1%)	59 (3.6%)	187 (3.9%)	408 (4.5%)	626 (4.0%)	0.177
Lifestyle eGFR Category 4	111 (2.8%)	10 (4.5%)	11 (2.2%)	28 (2.8%)	62 (2.7%)	0.354
Metformin	108234 (68.7%)	30389 (77.1%)	29574 (75.1%)	26798 (68.0%)	21473 (54.5%)	<0.001
Metformin eGFR Category 1	37429 (79.2%)	20201 (79.2%)	12134 (80.4%)	4303 (77.4%)	791 (71.6%)	<0.001
Metformin eGFR Category 2	54434 (72.4%)	9224 (76.7%)	14534 (76.6%)	17394 (73.3%)	13282 (65.0%)	<0.001
Metformin eGFR Category 3	15754 (50.6%)	911 (55.0%)	2806 (58.1%)	4946 (54.2%)	7091 (45.6%)	<0.001
Metformin eGFR Category 4	617 (15.3%)	53 (23.7%)	100 (19.8%)	155 (15.6%)	309 (13.4%)	<0.001
Sulphonylureas/ Repaglinide	76869 (48.8%)	16450 (41.7%)	18871 (47.9%)	19996 (50.7%)	21552 (54.7%)	<0.001
Sulphonylureas/ Repaglinide eGFR Category 1	21684 (45.9%)	10717 (42.0%)	7409 (49.1%)	2923 (52.6%)	635 (57.5%)	<0.001
Sulphonylureas/ Repaglinide eGFR Category 2	38060 (50.6%)	5012 (41.7%)	9090 (47.9%)	12229 (51.5%)	11729 (57.4%)	<0.001
Sulphonylureas/ Repaglinide eGFR Category 3	15742 (50.6%)	673 (40.7%)	2229 (46.2%)	4516 (49.5%)	8324 (53.6%)	<0.001
Sulphonylureas/ Repaglinide eGFR Category 4	1383 (34.3%)	48 (21.4%)	143 (28.3%)	328 (33.0%)	864 (37.5%)	<0.001
Acarbose	4613 (2.9%)	937 (2.4%)	1099 (2.8%)	1233 (3.1%)	1344 (3.4%)	<0.001
Acarbose eGFR Category 1	1267 (2.7%)	621 (2.4%)	440 (2.9%)	176 (3.2%)	30 (2.7%)	0.003
Acarbose eGFR Category 2	2096 (2.8%)	251 (2.1%)	454 (2.4%)	720 (3.0%)	671 (3.3%)	<0.001

(*Continued*)

Antihyperglycemic treatments	Age quartiles					p
	All	<62 years	62-69 years	70-75 years	>75 years	
	n=157595	n=39407	n=39394	n=39404	n=39390	
Acarbose eGFR Category 3	1120 (3.6%)	58 (3.5%)	191 (4.0%)	311 (3.4%)	560 (3.6%)	0.633
Acarbose eGFR Category 4	130 (3.2%)	7 (3.1%)	14 (2.8%)	26 (2.6%)	83 (3.6%)	0.398
Glitazones	4280 (2.7%)	1250 (3.2%)	1263 (3.2%)	1090 (2.8%)	677 (1.7%)	<0.001
Glitazones eGFR Category 1	1437 (3.0%)	803 (3.1%)	462 (3.1%)	150 (2.7%)	22 (2.0%)	0.087
Glitazones eGFR Category 2	2056 (2.7%)	393 (3.3%)	651 (3.4%)	630 (2.7%)	382 (1.9%)	<0.001
Glitazones eGFR Category 3	725 (2.3%)	53 (3.2%)	143 (3.0%)	290 (3.2%)	239 (1.5%)	<0.001
Glitazones eGFR Category 4	62 (1.5%)	1 (0.4%)	7 (1.4%)	20 (2.0%)	34 (1.5%)	0.401
Insulin	48831 (31.0%)	10754 (27.3%)	11017 (28.0%)	12489 (31.7%)	14571 (37.0%)	<0.001
Insulin eGFR Category 1	12007 (25.4%)	6719 (26.3%)	3603 (23.9%)	1410 (25.4%)	275 (24.9%)	<0.001
Insulin eGFR Category 2	20693 (27.5%)	3121 (26.0%)	4951 (26.1%)	6573 (27.7%)	6048 (29.6%)	<0.001
Insulin eGFR Category 3	13375 (43.0%)	750 (45.3%)	2109 (43.7%)	3813 (41.8%)	6703 (43.2%)	0.074
Insulin eGFR Category 4	2756 (68.4%)	164 (73.2%)	354 (70.0%)	693 (69.7%)	1545 (67.0%)	0.114

Absolute frequency (percentage) of each antihyperglycemic treatment across estimated glomerular filtration rate (eGFR) categories and age quartiles. The p values refer to significance of logistic mixed regression models for each treatment by age quartiles within each eGFR category.

Supplementary Table 2: Clinical characteristics of the whole sample by anti-hyperglycemic treatment.

See Supplementary File 1
