# Supplementary material for: Antihyperglycemic treatment in patients with type 2 diabetes in Italy: the impact of age and kidney function
Source: Oncotarget. 2017 Jun 28;8(37):62039–48. doi: 10.18632/oncotarget.18816 (PMC5617484; doi:10.18632/oncotarget.18816)
Supplement: Supplementary file 2 [file oncotarget-08-62039-s002.docx]

**Supplementary** **Table 2. Clinical characteristics of the whole sample by anti-hyperglycemic treatment**

|  | **Lifestyle** | **Metformin only** | **Metformin plus insulin** | **Oral hypoglycemic agents** | **Insulin** |  |
| --- | --- | --- | --- | --- | --- | --- |
|  | **n=8229** | **n=29821** | **n=22370** | **n=70714** | **n=26461** | **p** |
| Male sex (n) | 4988 (60.6%) | 17447 (58.5%) | 11597 (51.8%) | 40573 (57.4%) | 14685 (55.5%) | <0.001 |
| Age (years) | 67±11 | 65±10 | 67±10 | 68±11 | 70±12 | <0.001 |
| Former smokers (n) | 1563 (31.5%) | 5548 (30.3%) | 3436 (28.0%) | 11397 (29.3%) | 3931 (28.1%) | <0.001 |
| Current smokers (n) | 724 (14.6%) | 3226 (17.6%) | 2129 (17.4%) | 6476 (16.6%) | 2238 (16.0%) | <0.001 |
| Age at DM diagnosis (years) | 62±11 | 58±11 | 50±11 | 57±11 | 53±14 | <0.001 |
| Known duration of diabetes (years) | 5±6 | 6±6 | 16±9 | 11±8 | 17±11 | <0.001 |
| HbA1c (%) | 6.3±0.8 | 6.7±1.0 | 8.0±1.4 | 7.1±1.2 | 7.7±1.5 | <0.001 |
| BMI (Kg/m^2^) | 28±5 | 30±5 | 31±6 | 29±5 | 29±5 | <0.001 |
| BMI Men (Kg/m^2^) | 28.2±4.2 | 29.5±4.7 | 30.1±5.0 | 29.1±4.7 | 28.5±4.8 | <0.001 |
| BMI Women (Kg/m^2^) | 29±5 | 31±6 | 31±6 | 30±6 | 29±6 | <0.001 |
| Waist circumference (cm) | 100±12 | 104±12 | 106±13 | 103±13 | 102±13 | <0.001 |
| Waist circumference Men (cm) | 102±11 | 105±12 | 107±12 | 105±12 | 103±13 | <0.001 |
| Waist circumference Women (cm) | 98±12 | 102±12 | 106±13 | 102±13 | 101±14 | <0.001 |
| Triglycerides (mg/dL) | 124±74 | 136±83 | 147±108 | 136±87 | 136±95 | <0.001 |
| HDL (mg/dL) | 53±15 | 50±14 | 49±14 | 50±14 | 50±16 | <0.001 |
| HDL Men (mg/dL) | 50±14 | 47±13 | 45±13 | 47±13 | 47±15 | <0.001 |
| HDL Women (mg/dL) | 57±15 | 54±14 | 53±15 | 54±14 | 55±17 | <0.001 |
| LDL (mg/dL) | 111±33 | 103±33 | 97±34 | 101±33 | 100±34 | <0.001 |
| Non-HDL (mg/dL) | 136±36 | 130±37 | 125±38 | 127±37 | 127±39 | <0.001 |
| Systolic BP (mmHg) | 135±18 | 136±18 | 139±19 | 137±18 | 137±19 | <0.001 |
| Diastolic BP (mmHg) | 78±9 | 79±9 | 78±9 | 78±9 | 76±10 | <0.001 |
| Pulse pressure (mmHg) | 57±15 | 57±15 | 62±17 | 59±16 | 60±17 | <0.001 |
| Albuminuria (n) | 1686 (20.5%) | 6699 (22.5%) | 7734 (34.6%) | 19303 (27.3%) | 9965 (37.7%) | <0.001 |
| Microalbuminuria (n) | 1445 (17.6%) | 5705 (19.1%) | 5919 (26.5%) | 15605 (22.1%) | 7127 (26.9%) | <0.001 |
| Macroalbuminuria (n) | 241 (2.9%) | 994 (3.3%) | 1815 (8.1%) | 3698 (5.2%) | 2838 (10.7%) | <0.001 |
| Serum creatinine (mg/dL) | 0.95±0.47 | 0.88±0.39 | 0.92±0.47 | 0.95±0.48 | 1.22±0.78 | <0.001 |
| eGFR (mL/min/1.73 m^2^) | 78±19 | 83±17 | 78±20 | 77±20 | 65±26 | <0.001 |
| Retinopathy (n) | 180 (2.2%) | 1419 (4.8%) | 6364 (28.4%) | 7353 (10.4%) | 6934 (26.2%) | <0.001 |
| Antihypertensive treatment (n) | 5168 (62.8%) | 20458 (68.6%) | 16971 (75.9%) | 49982 (70.7%) | 19845 (75.0%) | <0.001 |
| Treatment with ACE-Is/ARBs (n) | 4262 (51.8%) | 17524 (58.8%) | 14962 (66.9%) | 43012 (60.8%) | 16061 (60.7%) | <0.001 |
| Lipid-lowering treatment (n) | 3878 (47.1%) | 16532 (55.4%) | 14400 (64.4%) | 40972 (57.9%) | 14908 (56.3%) | <0.001 |
| Treatment with statins (n) | 3653 (44.4%) | 15244 (51.1%) | 13176 (58.9%) | 37582 (53.1%) | 13687 (51.7%) | <0.001 |
| Aspirin (n) | 1287 (15.6%) | 5738 (19.2%) | 6006 (26.8%) | 15891 (22.5%) | 6362 (24.0%) | <0.001 |
| Q Score | 31±7 | 31±7 | 27±8 | 30±8 | 28±9 | <0.001 |
| eGFR<60 mL/min/1.73 m^2^ | 1391 (16.9%) | 3325 (11.1%) | 4288 (19.2%) | 14319 (20.2%) | 11843 (44.8%) | <0.001 |
| HbA1c ≥7% | 1229 (15.0%) | 9364 (32.0%) | 16945 (76.7%) | 34809 (49.9%) | 17542 (67.4%) | <0.001 |
| Total cholesterol (mg/dL) | 188±38 | 180±38 | 174±39 | 177±38 | 177±41 | <0.001 |
| Triglycerides ≥150 mg/dl (n) | 1840 (24.0%) | 8561 (30.5%) | 7317 (34.7%) | 20439 (30.9%) | 7310 (30.0%) | <0.001 |
| HDL <40M <50F mg/dL (n) | 2036 (26.9%) | 9190 (33.2%) | 8525 (41.2%) | 23321 (35.9%) | 9036 (37.8%) | <0.001 |
| LDL ≥100 mg/dL (n) | 4610 (60.9%) | 14041 (51.1%) | 8625 (42.0%) | 30954 (47.8%) | 11065 (46.5%) | <0.001 |
| Blood Pressure ≥140/85 mmHg (n) | 3512 (51.1%) | 13250 (51.8%) | 11154 (57.6%) | 32285 (54.1%) | 11261 (51.3%) | <0.001 |
| Treatment with fibrates (n) | 115 (1.4%) | 799 (2.7%) | 835 (3.7%) | 2212 (3.1%) | 627 (2.4%) | <0.001 |
| Metformin (n) | 0 (0.0%) | 29821 (100.0%) | 22370 (100.0%) | 56043 (79.3%) | 0 (0.0%) | - |
| Sulphonylureas/Repaglinide (n) | 0 (0.0%) | 0 (0.0%) | 11525 (51.5%) | 61709 (87.3%) | 3635 (13.7%) | - |
| Acarbose (n) | 0 (0.0%) | 0 (0.0%) | 712 (3.2%) | 3233 (4.6%) | 668 (2.5%) | - |
| Glitazones (n) | 0 (0.0%) | 0 (0.0%) | 208 (0.9%) | 3901 (5.5%) | 171 (0.6%) | - |
| Insulin (n) | 0 (0.0%) | 0 (0.0%) | 22370 (100.0%) | 0 (0.0%) | 26461 (100.0%) | - |

Mean±SD or absolute frequency (percentage). The p values refer to significance of mixed regression models (linear for continuous and logistic for categorical variables) with groups of anti-hyperglycemic treatment as dependent variables. Legend as in Table 1
